# Methods of competing risks flexible parametric modeling for estimation of the risk of the first disease among HIV infected men

**DOI:** 10.1186/s12874-020-0900-z

**Published:** 2020-01-29

**Authors:** Sahar Nouri, Mahmood Mahmoudi, Kazem Mohammad, Mohammad Ali Mansournia, Mahdi Yaseri, Noori Akhtar-Danesh

**Affiliations:** 1 Department of Epidemiology and Biostatistics, School of public health, Tehran University of Medical Sciences, Tehran, Iran; 2 School of Nursing, Faculty of Health Sciences, McMaster University, Hamilton, Canada; 3 Department of Health Research Methods, Evidence, and Impact, McMaster University, Hamilton, Canada

**Keywords:** Competing risks, Flexible parametric models, Multicenter AIDS cohort study, Hazard function, Subdistribution Hazard function, Risk

## Abstract

**Background:**

Patients infected with the Human Immunodeficiency Virus (HIV) are susceptible to many diseases. In these patients, the occurrence of one disease alters the chance of contracting another. Under such circumstances, methods for competing risks are required. Recently, competing risks analyses in the scope of flexible parametric models have risen to address this requirement. These lesser-known analyses have considerable advantages over conventional methods.

**Methods:**

Using data from Multi Centre AIDS Cohort Study (MACS), this paper reviews and applies methods of competing risks flexible parametric models to analyze the risk of the first disease (AIDS or non-AIDS) among HIV-infected patients. We compared two alternative subdistribution hazard flexible parametric models (SDH_FPM_1 and SDH_FPM_2) with the Fine & Gray model. To make a complete inference, we performed cause-specific hazard flexible parametric models for each event separately as well.

**Results:**

Both SDH_FPM_1 and SDH_FPM_2 provided consistent results regarding the magnitude of coefficients and risk estimations compared with estimations obtained from the Fine & Gray model, However, competing risks flexible parametric models provided more efficient and smoother estimations for the baseline risks of the first disease. We found that age at HIV diagnosis indirectly affected the risk of AIDS as the first event by increasing the number of patients who experience a non-AIDS disease prior to AIDS among > 40 years. Other significant covariates had direct effects on the risks of AIDS and non-AIDS.

**Discussion:**

The choice of an appropriate model depends on the research goals and computational challenges. The SDH_FPM_1 models each event separately and requires calculating censoring weights which is time-consuming. In contrast, SDH_FPM_2 models all events simultaneously and is more appropriate for large datasets, however, when the focus is on one particular event SDH_FPM_1 is more preferable.

## Background

There are a variety of possible subsequent outcomes other than Acquired Immunodeficiency Syndrome (AIDS) among HIV-infected patients [1–3]. When our primary focus is on the occurrence of each of these events separately in the presence of each other, the problem of competing risks arises [4, 5]. Competing risks occur when subjects can experience one or more events, which ‘compete’ with each other and occurrence of one event may prohibit observing the other events or modify their chances of occurrence. With the occurrence of non-AIDS diseases or death prior to AIDS, the true survival time for developing AIDS would be unobservable (e.g., tuberculosis, a lung infection, can expedite progression to AIDS). In general, among HIV-infected patients, diseases can affect each other through immune system suppression. Treatment of one disease may reduce the chance of developing another disease [6–8]. One solution to assess an unbiased estimate of the risk of the event of interest is restricting the analysis to competing events where the follow up for a patient ends at the onset of the first event, and not focusing on multiple events in a patient [9–11]. In this scenario, traditional survival analysis presumes that only one event can occur is not valid and this calls for special methods for competing risks, [5, 12]. Using well-known Kaplan-Meier method, which considers each patient experiencing a competing event as censoring at the time of its occurrence, undermines an important assumption underlying this estimator: independent censoring. However, the subject who was censored due to failing in a competing event will never experience the event of interest as the first event and considering competing events to be independent censorings leads to biases and overestimates the probability of failure [5, 12, 13]. An appropriate measure to evaluate the probability of failure within a competing risk framework is the cumulative incidence function (CIF) [14–16]. It is defined as the probability of failure from the event of interest in an interval of time from the beginning of the study until a particular time when it is quite clear how many subjects experienced competing events.

To evaluate the effects of covariates in a competing risks analysis, model-based approaches on two important measures —the cause-specific hazard and subdistribution hazard— are more common. The difference between these two measures is in their risk sets. In the cause-specific hazard, patients who experienced a competing event will be excluded from the risk set for the event of interest and considered as censored. Modeling on cause-specific hazards provides causal effects of covariates on the hazard of the event of interest in a counterfactual world, where there are no competing events and patients can only fail from the event of interest [10, 11, 14, 16]. In contrast, in the cause-specific subdistribution hazard a patient experiencing a competing event will remain in the risk set until the end of the study because he can never experience the event of interest as the initial event. Modeling on subdistribution hazard creates the effect of covariates on the probability of failing in the event of interest at the presence of competing events which is a more realistic depiction of subjects who follow the event of interest and takes into account the chance of failing from other causes prior to the event of interest [5, 16–18]. The choice of modeling hazard or subdistribution hazard depends on the research goals. For etiological goals, to investigate the effect of covariates on the occurrence of a particular event, cause-specific hazard models are suitable. In contrast, for prognosis questions, to know what fraction of subjects at a specified time are at risk of experiencing the event of interest as the first event, considering the fact that they can experience a competing event as the initial event, subdistribution hazard models are preferable [5, 11, 12]. The two most popular models based on cause-specific hazards and subdistribution hazards are Cox and Fine & Gray regression models respectively [19, 20].

There are alternative approaches in modeling of competing risks based on the decomposition of the CIF (i.e., the joint distribution of event times and types of events) known as mixture and vertical models. Mixture models factorize the CIF as the product of the marginal distribution of event type and the conditional distribution of failure time given the type of the event. A multinomial regression model is used to assess the effects of covariates on the type of event and parametric or semi-parametric hazard regression models are used to evaluate their effects on the conditional failure times [21–24]. In contrast, vertical models decompose the joint distribution of event times and types of events as the product of the marginal distribution of time of failure (ignoring the type of event) and conditional distribution of event type given the time of failure, which provides relative hazards [25]. With that said, these models have some difficulties in their estimations, and interpretation of their results and are not practically well-developed [26].

One important issue with Cox or Fine & Gray models is that the baseline hazard or subdistribution hazard functions remain unspecified and are not estimated parametrically [4, 9, 20]. Their use of partial likelihood functions and inferences are limited to the relative rate of the two hazards or subdistribution hazards. To capture the shape of baseline functions more accurately, the use of parametric models in competing risks modeling, and generally for survival analysis, are preferred. The ordinary forms of parametric models (e.g., Weibull and log-logistic have a constraint of linear association between transformations of the survival function and log time [27]. Then the flexibility of the models to fit adequately on the data is limited. So, bias estimations and inaccurate predictions result. In recent years, the application of flexible parametric models in the competing risks modeling for both cause-specific hazard and subdistribution hazard approaches have been proposed [28–30]. Flexible parametric models are an extension of parametric models and can be defined on a wide class of different scales (e.g., hazard scale, odds scale or probit). They model a transformation of baseline survival functions on the log time scale using Natural Cubic Splines (NCSs) instead of linear functions and have substantial capabilities in assessing parametric estimates of the absolute measure of hazard or subdistribution hazard of the event of interest at each time point [31, 32].

The primary aim of this paper is to apply two alternative subdistribution hazard flexible parametric models to the HIV-infected men population and compare these models with the Fine & Gray model as a standard model in competing risks analysis. We have identified the competing risks as the first event —*AIDS, non-AIDS, and death prior to AIDS or non-AIDS diseases*— in an HIV-infected male population and evaluated the covariates that are associated with the risks of these outcomes. Furthermore, we implemented a cause-specific hazard flexible parametric model to investigate the direct (causal) and indirect (noncausal) effects of covariates on the risk of the competing events. In the next section, we present a description of the Multicenter AIDS Cohort Study (MACS). In the third section, the association of the cumulative incidence function with the hazard and subhazard functions is explained. In the fourth section, the Competing risks flexible parametric models (CR_FPM_s) for multiple types of events are reviewed. In the fifth section, the CR_FPM_s are applied to the MACS dataset and predictions for the risks are obtained. The last two sections contain results and conclusions.

## Study description

### Study population and patient selection

Multi Centre AIDS Cohort Study (MACS) is a 30–year prospective study of HIV infection among homosexual or bisexual men (HBM) who were 18 years or older with no prior AIDS-defining illness. MACS began in 1984 at four US sites; Chicago, Illinois; Baltimore, Maryland; Pittsburgh, Pennsylvania; and Los Angeles, California. It has multiple patient recruitments. The first recruitment, which consists of 4954 HIV-infected and uninfected HBM was conducted in 1984–1985. In 1987–1990, recruitment was reopened and 668 HBM were enrolled. During 2001–2003 another 1350 HBM were enrolled. Another MACS expansion commenced at the beginning of 2010 and 371 HBM were recruited until April 2014. HIV-related symptoms, demographic characteristics, blood specimens, and behavioral history at each 6-month follow-up visit were collected. Among 7232 HBM, seroconverter patients were selected and patients with a non-AIDS disease before seroconversion were excluded from the study. This analysis includes 674 seroconverter or prevalent patients with a known visit of seroconversion.

### Outcomes and covariates

Study outcomes were determined as the time duration from seroconversion to the occurrence of the first event. The midpoint of the last negative and the first positive visits was used as the time of seroconversion. The primary event was the occurrence of AIDS without evidence of a non-AIDS disease before. The secondary event was time to a non-AIDS disease prior to AIDS. Non-AIDSs included the following diseases: kidney, liver, cardiovascular, cerebrovascular diseases; lung infection, bacteremia, septicemia; malignancies, neurologic; cancers —all cancers excluding Kaposi sarcoma, lymphoma, and invasive cervical cancer. Since the occurrence of death precludes observing AIDS or non-AIDS diseases, we considered unrelated death as the third outcome. These types of deaths may occur for reasons unrelated to AIDS or non-AIDS diseases (e.g., cerebral artery occlusion). Other patients who were lost to follow-up or did not experience any failure event at the end of the study (i.e., April 2014) were censored. Such variables as MACS recruitment calendar years, age at seroconversion time, laboratory results including the number of positive CD4 cell counts, CD8 cell counts, white blood cells, red blood cells and platelets at baseline were considered based on the expert knowledge and previous studies on HIV/AIDS [6, 7, 33, 34]. Measurements obtained at the first positive visit are referred to as ‘baseline’. We used categorical covariates instead of continuous to have perceptible clinical interpretations. The cut points were determined based on clinical considerations and previous studies on the MACS data [33–38]. Sparse groups were integrated with adjacent categories. The study follow-up time was restricted to 15 years from HIV positive diagnosis to exclude non-AIDS diseases or causes not related to HIV infection or death related to aging.

## Relations of risk with Hazard and subhazard functions

Cause-specific CIF is a measure of absolute risk and defined as the probability of failure from k^th^ (k = 1 … K) cause by time t while being at risk of failing from other competing events [9]. So, we have:

1 $$ {F}_k(t)=p\left(T\le t, event=k\right)={\int}_0^t{h}_k^{cs}(u).S(u) du $$

In the above equation, *F*_*k*_(*t*) is the CIF function, *T* is the survival time i.e. the minimum of the true survival time and censoring time, ${h}_k^{cs}(u)$ is the cause-specific hazard functions at time *u* ≤ *t*. *S* is the overall survival function and is defined as.

` $$ S(u)=\exp \left\{-\sum \limits_{k=1}^K{\int}_0^u{h}_k^{cs}\ (v) dv\right\}. $$

Equation (1) implies that the risk of the event of interest is the combination of its hazard (${h}_k^{cs}$) and the chance that competing events get to the event of interest to occur as the first event (*S*). An increase in the hazard of competing events will lower the risk occurrence of the event of interest as the first event by decreasing the overall survival function. In other words, in a competing risks framework, a covariate can increase the risk of the event of interest directly via increasing the hazard of the event or indirectly by decreasing the hazards of competing events. So, a discrepancy between the hazard and risk of the event of interest may be observed. The magnitude of this discrepancy depends on the severity of competing risks. The stronger the competing risk, the greater the discrepancy [10, 12, 14]. The cause-specific hazard is the instantaneous failure rate from a particular event among patients who did not experience any prior competing events and has the form of

$$ {h}_k^{cs}(t)=\underset{\Delta  t\to 0}{\mathrm{Lim}}\left\{\frac{p\left(t\le T<t+\Delta  t, event=k|T\ge t\right)}{\Delta  t}\right\}. $$

In contrast, the cause-specific subdistribution hazard is defined as

$$ {h}_k^{sd}(t)=\underset{\Delta  t\to 0}{\mathrm{Lim}}\left\{\frac{p\left(t<T\le t+\Delta  t, event=k|T>t\cup \left(T<t\cap K\ne k\right)\right)}{\Delta  t}\right\}. $$

Although this definition of the risk set is not practically meaningful because the patient does not actually remain in the risk set to experience the event of interest as the first event; however, this can lead to the following direct relationship between subdistribution hazard functions and corresponding cause-specific CIFs:

2 $$ {F}_k(t)=1-\exp \left\{-{\int}_0^t{h}_k^{sd}(u) du\right\} $$

So, modeling on cause-specific subdistribution hazard will show the associations between covariates on the cause-specific CIF. The subdistribution hazard-based models use the information that the occurrence of competing events gives about the event of interest. That is, the event of interest never occurs as the first event if a competing event has already occurred. So, whenever a competing event occurs the chance of occurrence of the event of interest as the first event will be reduced. However, to investigate whether an association is direct or indirect the effect of the covariate on all cause-specific hazards should be assessed.

## Models

### Cause-specific Hazard flexible parametric models

The CSH_FPM_ performs a flexible parametric model for each type of event separately considering competing events as censoring. It regresses the cause-specific log cumulative hazard function on a Natural Cubic Splines (NCSs) function of the log of time that is

3 $$ \ln {H}_k^{cs}\left(t|{X}_k\right)=\ln {H}_{0k}(t)+{X}_k{\beta}_k={NCS}_k\left\{ lnt;{\gamma}_k,{d}_{0k}\ \right\}+{X}_k{\beta}_k $$

Where *H*^*cs*^(*t*|*X*) is the cause-specific cumulative hazard function for event k with matrix *X*_*k*_ of covariates at time *t*, *H*_0*k*_ (*t*) is the cause-specific baseline cumulative hazard function, *β*_*k*_ is a vector of covariates coefficients and *NCS*_*k*_{*lnt*; *γ*_*k*_, *d*_0*k*_} is a natural cubic spline function of *ln*(*t*) with *d*_0_ knots and parameters *γ* for event k. The number and position of knots in the spline function determine the complexity of the baseline cumulative hazard function. However, sensitivity analysis showed that they have little effect on the model fitting and there is no need for optimal selection to have a good fit [31]. Model [3] has the capability of carrying time dependent effects of covariates, for handling non-proportional hazards (non-PH) form, easily through incorporating the interactions of covariates with spline functions [32].

Instead of separate CSH_FPM_s, Hinchliffe and Lambert introduced a unified CSH_FPM_ on stacked data based on the Lunn-McNeil approach [28, 39]. In the stacked data, each patient has one row of observations for each particular competing event to have the opportunity of failing in that event. For each event type, an indicator variable would be added to the data. An additional indicator variable also would be created to identify the type of event for each patient. The unified CSH_FPM_ is fitted for all competing events simultaneously and parameters related to each competing event are jointly estimated [40]. Different baseline hazard functions are considered for each cause of failure. This approach has the capability of considering shared covariate effects for all competing events. If knot positions in the unified model are the same as those in separate CSH_FPM_s, covariate estimations of this model would be equivalent to those obtained from separate models [40, 41].

### Subdistribution Hazard flexible parametric models (SDH_FPM_)

In the SDH_FPM_, the effects of covariates directly model on the cause-specific log cumulative subdistribution hazard functions using NCSs as follows:

4 $$ \ln {H}_k^{sd}\left(t|{X}_k\right)=\ln \left(-\ln \left(1-{F}_k\left(t|{X}_k\right)\right)\right)={NCS}_k\left\{ lnt;{\gamma}_k,{d}_{0k}\ \right\}+{X}_k{\beta}_k $$

Lambert et al. proposed a parametric version of the Fine & Gray model [29]. Patients who experienced a competing event are kept in the risk set and have the chance of being censored before the end of the follow-up. The survival time is defined as the minimum of censoring time and true survival time. So, the censoring probabilities should be calculated and incorporate in the likelihood function for obtaining an unbiased estimation of the subdistribution hazard for the event of interest [40]. Fine and Gray calculated the censoring probabilities non-parametrically and used inverse probabilities of censoring weights in the partial likelihood function [20]. In the Lambert model, the censoring distribution is estimated parametrically using FPMs. They used a weighted likelihood where weights are conditional probability of not being censored after experiencing a competing event which is time dependent because the censoring probability grows over time. In addition, they extended the Geskus approach to be able to estimate SHD_FPM_ using standard software of FPMs [42]. To achieve this, the follow up time after competing event would be split into a number of intervals and time dependent weights are applied to each interval. Then, standard packages for FPMs can be applied for the event of interest [29]. Lambert showed that there is no need to have a very fine number of splits and the bias of estimation is negligible. Like the Fine & Gray model, the Lambert model is fitted for each event of interest separately [29, 41].

As an alternative method, Mozumder et al. introduced a unified likelihood function to obtain a direct estimate of all cause-specific CIFs simultaneously using FPMs [30]. This model is also on the log cumulative subdistribution hazards scale (Eq. (4)). In this method, however, instead of using the censoring weights, the likelihood function is directly constructed based on subdistribution hazards and CIFs [30, 43]. We hereafter refer to the former subdistribution hazard model as SDH_FPM_1 and the latter as SDH_FPM_2.

## Statistical analysis

We performed a Complete Case Analysis (CCA), 629 of 674 patients, and assumed the data were Missed Completely at Random (MCAR). However, Multiple Imputations (MI) using the multivariate normal regression method with 10 imputed data sets for missing data were performed to explore the sensitivity of the inferences to departures from MCAR assumptions.

The possibility of a reduction in the number of initial set of covariates, generated based on the expert (background) knowledge, was explored. We used *transcan* function in R, which transforms covariates while imputing missing values of them [44]. The transformation for each covariate is conducted using canonical variates in a way that the covariate has the maximum correlation with the optimum linear combinations of other covariates. The number of knots for baseline hazard and subhazard functions were determined using the Akaike Information Criterion (AIC) statistic. We used main effects of covariates and started with five degrees of freedom and identified the complexity of models based on the lowest value of AIC. Internal and boundary Knots for NCSs of each competing event separately located in their equally spaced centiles and the first and last event times respectively. After determining the optimal number of knots for each model, a forward stepwise regression method was performed to build final models [45]. However, we did not consider variable selection on CD4 cell count due to its important role in HIV/AIDS studies. In SDH_FPM_1, for patients experiencing a competing event the time was split every .1-year. The time dependent censoring weights were estimated through fitting an FPM on the initial set of covariates, with three degrees of freedom. The CSH_FPM_s were conducted for each event separately.

All significant effects were detected using the Likelihood Ratio (LR) tests at a 5% level. The findings are summarized with hazard and subdistribution hazard ratios and 95% Confidence Intervals (CI). Statistical analyses were performed in STATA (StataCorp. 2017. Stata Statistical Software: Release 15. College Station, TX: StataCorp LLC.) and R (Version 3.5.2) [46].

## Results

The descriptive statistics for patients in the study are reported in Table 1. Patients who were diagnosed with AIDS as the first event tended to be younger, have more prevalence in 1984–85 and 1987–90 recruitments, lower CD4, higher CD8, and higher RBC in comparison with patients who were diagnosed with non-AIDS diseases as the first event. The results of *transcan* function showed that none of the covariates selected by the expert (background) knowledge had strong correlations with the others (refer to Additional file 1: Table S1). The AICs and BICs in all three CSH_FPM_, SDH_FPM_1 and SDH_FPM_2 with 3, 1, 1 knot(s) for AIDS, nonAIDS, and death respectively, had minimum values. For all CR_FPM_s, Interactions between the age with ln(t) on non-AIDS diseases was statistically significant (LR test *P*-values = .05, .021 and .024, for CSHFPM, SDHFPM1 and SDHFPM2, respectively). More complicated time dependent effects of age had been assessed. However, ln (t) had the lowest AIC and BIC among them. Table 2 presents the results of multivariable SDH_FPM_1, SDH_FPM_2, and Fine & Gray model, which show the effects of covariates on the subdistribution hazards or equivalently on the risk of the first observed event. There was a high agreement between estimated subhazard ratios obtained from Fine & Gray and two SDH_FPM_s. The results of performing CSH_FPM_ also show the effects of covariates on the hazard of each event separately (refer to Table 2). For simplicity, we refer to 1984–85 and 1987–90 MACS recruitments as period 1 and 2001–3 and 2010 recruitments as period 2. The rest of this section is concentrated on the results of SDH_FPM_1 and CSH_FPM_. However, due to the fair agreement between models, the SDH_FPM_2 would have the same interpretation. The interpretation of subdistribution hazard ratios is not often appealing. It should be noted that their magnitudes are not equivalent to the effect of covariates on the risk of the event of interest. However, they contain information about the significance and direction of the effects of covariates on the risk of the event of interest [47]. The results of analyses on the multiply imputed data sets were almost identical to CCA (refer to Appendix).

**Table 1:** Baseline Characteristics of Seroconverter HBM in the MACS Data

Variables	^*^patients	AIDS (*N* = 267)	Non-AIDS (*N* = 156)	Death (*N* = 25)
MACS recruitment	*N* = 674			
1984–85 & 1987–90	581(86.20)	266(99.63)	132(84.62)	23(92.00)
2001–3 & 2010	93(13.80)	1(0.37)	24(15.38)	2(8.00)
Age at diagnosis	*N* = 667			
***<***40	473(70.91)	205(77.36)	94(60.26)	14(58.33)
**≥**40	194(29.09)	60(22.64)	62(39.74)	10(41.67)
Baseline CD4, per μl	*N* = 629			
< 350	51(8.11)	22(8.84)	9(6.04)	0(0.00)
350–500	102(16.22)	47(18.88)	16(10.74)	6(31.58)
**≥**500	476(75.68)	180(72.29)	124(83.22)	13(68.42)
Baseline CD8, per μl	N = 629			
< 500	91(14.47)	45(18.07)	17(11.41)	4(21.05)
500–1000	337(53.58)	129(51.81)	79(53.02)	12(63.16)
**≥**1000	201(31.96)	75(30.12)	53(35.57)	3(15.79)
Baseline WBC, per μl	*N* = 663			
< 5000	191(28.81)	78(29.43)	39(25.32)	10(43.48)
**≥**5000	472(71.19)	187(70.57)	115(74.68)	13(56.52)
Baseline RBC, ***×*** 10^5^, per μl	N = 667			
<4.5	90(13.49)	25(9.43)	31(19.87)	1(4.17)
**≥**4.5	577(86.51)	240(90.57)	125(80.13)	23(95.83)
Baseline platelets, ***×*** 10^3^, per μl	*N* = 660			
< 150	40(6.06)	10(3.77)	9(5.92)	6(26.09)
150–250	347(52.58)	127(47.92)	90(59.21)	8(34.78)
**≥**250	273(41.36)	128(48.30)	53(34.87)	9(39.13)

*Differences between the number of patients and the sum of the AIDS, Non-AIDS and Death columns indicate the number of censored patients

**Table 2:** Cause-Specific and Subdistribution Hazard Ratios Estimated from the Cause-Specific Hazard (CSH_FPM_) and Cause-Specific Subdistribution Hazard models (SDH_FPM_1, SDH_FPM_2 and Fine and Gray)

Event	Variable	SDH_FPM_1	SDH_FPM_2	Fine&Gray Model	CSH_FPM_
		_SD_HR (95% CI)	_SD_HR (95% CI)	_SD_HR (95% CI)	_CS_HR (95% CI)
AIDS	MACS recruitment				
	1984–85 & 1987–90	Reference	Reference	Reference	Reference
	2001–3 & 2010	.031(.004–.22)	.035(.005–.25)	.030(.004–.21)	.039(.005–.28)
	*LR test	<.0001	<.0001		.001
	Age at diagnosis				
	<40	Reference	Reference	Reference	Reference
	≥40	.69(.51–.93)	.69(.52–.93)	.70(.51–0.96)	.87(.64–1.18)
	LR test	.017	.016		.38
	Baseline CD4				
	< 350	1.83 (1.16–2.90)	1.70(1.10–2.63)	1.82(1.14–2.90)	1.86(1.19–2.90)
	350–500	1.38(.99–1.93)	1.32(.96–1.83)	1.38(1.00–1.91)	1.33(.96–1.84)
	≥ 500	Reference	Reference	Reference	Reference
	LR test	.016	.038		.016
Non-AIDS	MACS recruitment				
	1984–85 & 1987–90	Reference	Reference	Reference	Reference
	2001–3 & 2010	3.33(2.04–5.46)	3.57(2.22–5.75)	3.18(1.90–5.35)	2.45(1.51–3.99)
	LR test	<.0001	<.0001		.0009
	Age at diagnosis				
	<40	Reference	Reference	Reference	Reference
	≥40	2.88(1.93–4.31)	3.57(2.21–5.77)	2.51(1.72–3.68)	3.72(2.34–5.90)
	LR test	<.0001	<.0001		<.0001
	Baseline CD4				
	< 350	1.13(.56–2.32)	.84(.43–1.66)	1.11(.56–2.23)	1.32(.65–2.68)
	350–500	.58(.30–1.13)	.58(.30–1.11)	.57(.29–1.12)	.68(.35–1.32)
	≥ 500	Reference	Reference	Reference	Reference
	LR test	.19	.42		.33
Death	Age at diagnosis				
	<40	Reference	Reference	Reference	Reference
	≥40	2.95(1.07–8.15)	3.28(1.19–8.99)	2.91(1.05–8.04)	3.71(1.34–10.28)
	LR test	.037	.021		.012
	Baseline CD4				
	< 350	–	–	–	–
	350–500	1.79(.57–5.63)	1.91(.62–5.91)	1.77(.57–5.54)	2.04(.65–6.41)
	≥ 500	Reference	Reference	Reference	Reference
	LR test	.17	.13		.18

*LR test is the Likelihood Ratio test for evaluating the effect of each covariate in the multivariable SDH_FPM_1, SDH_FPM_2, and CSH_FPM_

### Associations of MACS recruitments with the risk of AIDS prior to non-AIDS diseases, non-AIDS prior to AIDS and unrelated death

The results showed that the hazard of AIDS occurrence in period 2 declined to about 96% compared to period 1 (*P*-value = .002). In addition, the hazard of non-AIDS diseases among patients of period 2 was 2.45 times that of period 1 (*P*-value = .002). By increasing the hazard of non-AIDS diseases in period 2, fewer patients remained at risk to experience AIDS prior to non-AIDS diseases compared to period 1. Consequently, the risk of observing AIDS as the first event was lower in period 2 compared to period 1 (*P*-value = .0001). In a similar way, the risk of observing a non-AIDS disease prior to AIDS increased in period 2 compared to period 1 (P-value <.0001). Therefore, there were direct and indirect associations between the MACS recruitments and the risk of AIDS and non-AIDS. MACS recruitments were not associated with the hazard and risk of unrelated death.

### Associations of age at HIV diagnosis with the risk of AIDS prior to non-AIDS diseases, non-AIDS prior to AIDS and unrelated death

Age at HIV diagnosis was not associated with the hazard of AIDS (*P*-value = .38). However, the risk of AIDS prior to a non-AIDS disease in the ≥ 40 age group decreased by 31% compared to 40 (P-value = .017). This discrepancy between the hazard and risk of AIDS indicated an indirect effect of age on the risk of AIDS that came from the higher hazard rate of non-AIDS diseases and unrelated death among the older age group. The effects of age groups on both the hazard and risk of non-AIDS diseases, controlling other covariates, were time dependent and the average effect of the ≥ 40 group on the hazard of non-AIDS diseases was about 3.7 times that of < 40 group (*P*-value <.0001 The risk of non-AIDS diseases and unrelated death was higher among ≥ 40 age group (P-value <.0001, .039 respectively)**.** As a result, fewer patients in the ≥ 40 age group remained exposed to experience AIDS prior to non-AIDS diseases. These findings also indicated that age at diagnosis had direct effects on the risks of non-AIDS and unrelated death.

### Associations of CD4 levels with the risk of AIDS prior to non-AIDS diseases, non-AIDS prior to AIDS and unrelated death

Hazard of AIDS increased about 83% for CD4 levels < 350 compared to CD4 levels≥ 500 (*P*-value = .018). The risk of AIDS for patients with CD4 cells < 350 reached statistical significance comparing to patients with ≥ 500 (P-value = .027). Levels of CD4 had no significant association with the hazards and risks of non-AIDS diseases. Hence, the effect of CD4 levels on the risk of AIDS was quite direct. Levels of CD4 had no significant associations with hazards and risks of non-AIDS diseases and unrelated death. Hence, the effect of CD4 levels on the risk of AIDS was quite direct.

**Fig. 1:**
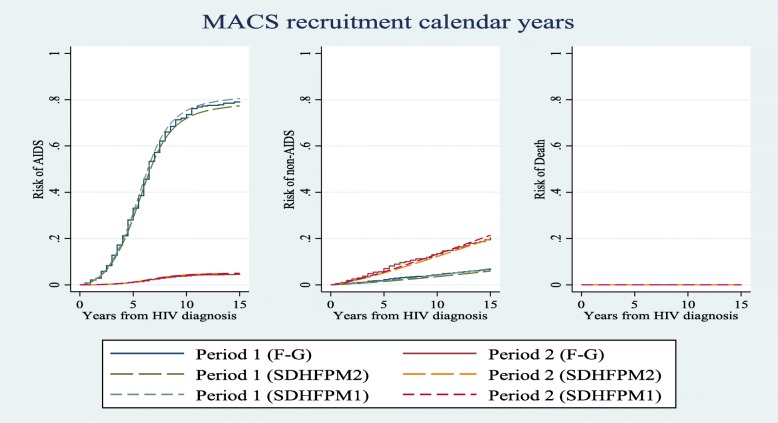
Comparisons of estimated risks of observing AIDS, non-AIDS and unrelated death as the first events among two groups of recruitments (1984–85 & 1987–90 as period 1 and 2001–3 & 2010 as period 2) obtained from Fine & Gray and two alternative subdistribution hazard flexible parametric models. The nonproportionality of hazards and subdistribution hazards were assumed for the effect age on the risk of non-AIDS diseases. Other covariates were controlled at age < 40 and CD4 < 350

**Fig. 2:**
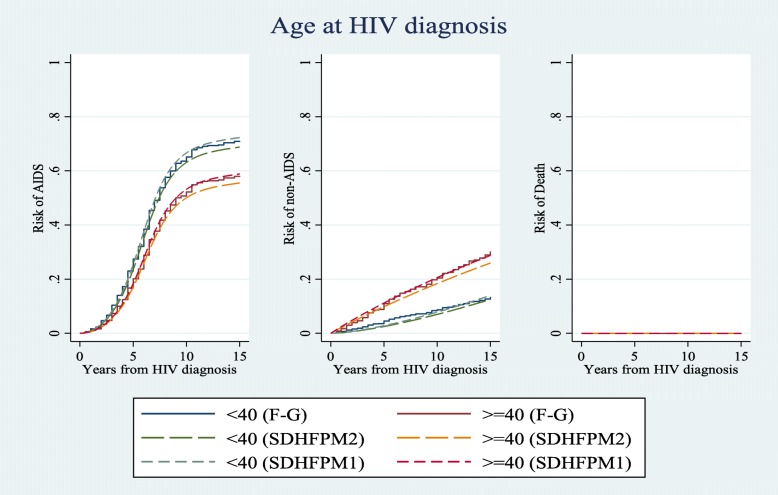
Comparisons of estimated risks of AIDS, non-AIDS and unrelated death as the first events among <40 and ≥ 40 age groups of seroconversion obtained from Fine & Gray and two alternative subdistribution hazard flexible parametric models. The nonproportionality of hazards and subdistribution hazards were assumed for the effect of age on non-AIDS diseases. Other covariates were controlled at period 1 (1984–85 & 1987–90) of MACS recruitments and CD4 < 350

**Fig. 3:**
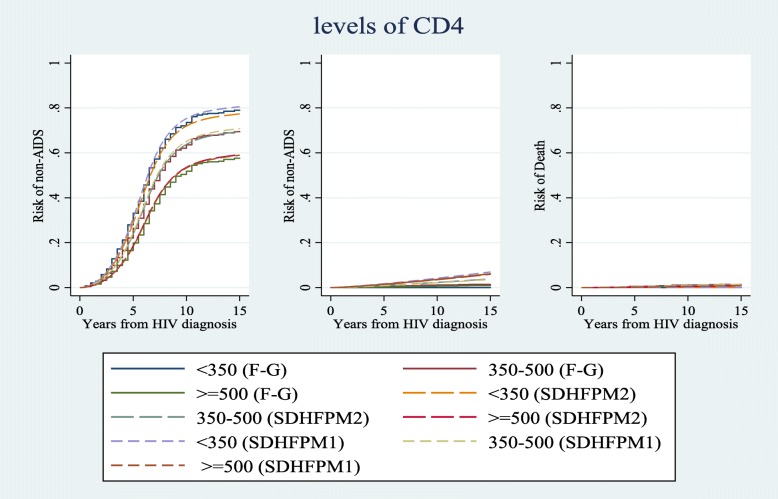
Comparisons of estimated risks of AIDS, non-AIDS and unrelated death as the first events among three levels of CD4 obtained from Fine & Gray and two alternative subdistribution hazard flexible parametric models. The nonproportionality of hazards and subdistribution hazards were assumed for the effect of age on non-AIDS diseases. Other covariates were controlled at period 1 (1984–85 & 1987–90) of MACS recruitments and age < 40

We compared risk estimations of competing events for different patterns of covariates in a 15-year time interval using all subdistribution hazard-based models (i.e. SDH_FPM_1, 2 and Fine & Gray). Figs. 1, 2, and 3 show that risk estimations obtained from SDH_FPM_1 and 2 were close to their corresponding values in the Fine & Gray model. The SDH_FPM_1 and 2 could accurately capture nonlinear trends of the risk of AIDS in different patterns of covariates and provide parametric estimations of them. For all patterns, the risk estimations of AIDS were dramatically higher than non-AIDS and risks of death were close to zero.

**Fig. 4:**
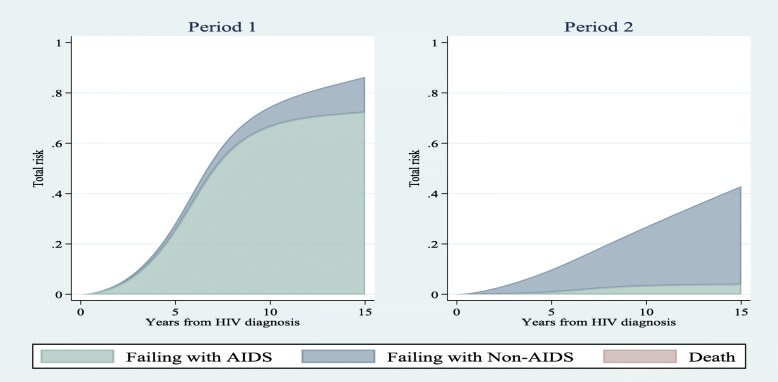
A comparison of stacked risks among MACS recruitments (1984–85 & 1987–90 as period 1 and 2001–3 & 2010 as period 2) for patients with age < 40 and CD4 < 350

**Fig. 5:**
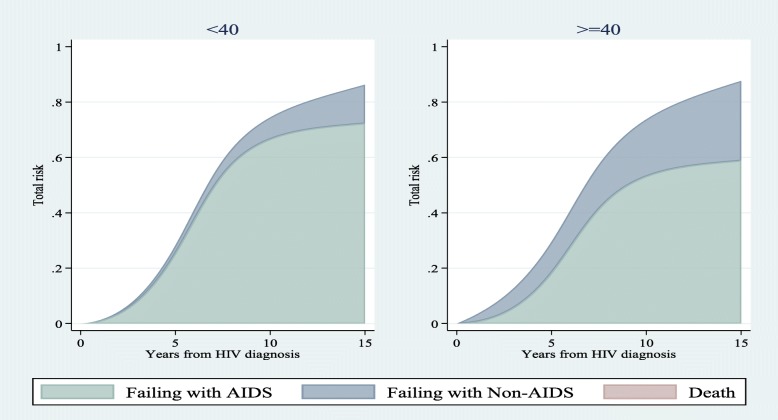
A comparison of stacked risks between two groups of age at diagnosis for patients in period 1 (1984–85 & 1987–90) of MACS recruitments with CD4 < 350

**Fig. 6:**
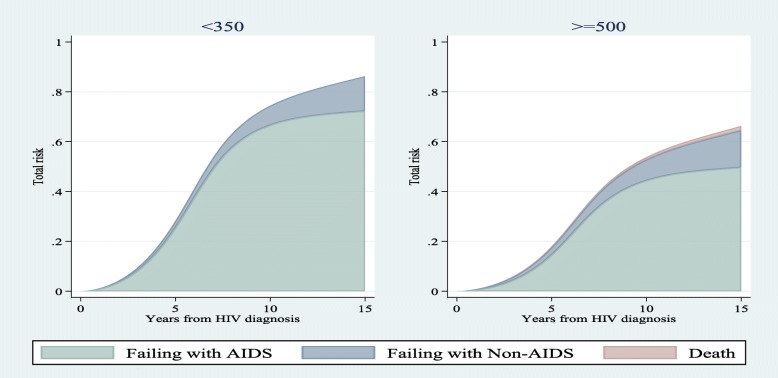
A comparison of stacked risks between two levels of baseline CD4 for patients in Period 1 (1984–85 & 1987–90) of MACS recruitments with age < 40

Figure 4, 5, and 6 demonstrate the stacked risk of the first event regardless of the type of the event and separated by the type of events. They compare risk estimations obtained from SDH_FPM_1 between different groups of patients. Fig. 4 compares the total risk of failure among two patients from periods 1 and 2 with controlling other covariates at the baseline level (the lowest level). The risk of experiencing the first event for a patient from period 1 was dramatically higher than period 2. In contrast, the risk of failure for a patient from period 2 was mostly related to non-AIDS diseases. Figs. *5* and *6* also show comparisons of stacked risks between two age groups at diagnosis and CD4 levels, respectively. Risk of failure for a patient < 40 years was almost equivalent to a patient ≥ 40 years. However, the probability that the failure is due to AIDS is higher for younger patients. The risk of experiencing a disease, mostly AIDS, for a patient with the lowest level of CD4 was higher than a patient with the highest level of CD4. The probability that a patient fails from death prior to experiencing a disease was near zero in all figures.

## Discussion

We have presented a comparison between competing risks flexible parametric and Fine & Gray models for the first disease among HIV positive men using the ongoing MACS data set. Two alternative subdistribution flexible parametric models were used. One of them was based on censoring weights and was performed for each competing event separately. The other one was based on direct estimations of the subdistribution functions and was performed simultaneously for all three competing risks. All the three models were similar regarding the estimations of covariate effects, their confidence intervals, and risk estimations. The main disadvantage of the Fine & Gray models is that the baseline risks remain unspecified and are assessed nonparametrically in a further step. They are, however, very flexible and do not make any assumptions on the shape of baseline functions. As the most important finding in our study, the SDH_FPM_s provided parametric estimations, which presented the data at hand accurately. They captured the complicated and nonlinear trend of the risk of AIDS using NCSs and provided smooth estimations. Among two subdistribution hazard-based flexible parametric models, SDH_FPM_2 is more straightforward to run than SDH_FPM_1 and can be directly implemented on the original data. In addition, calculating censoring weights and splitting the data in SDH_FPM_1 leads to intensive computations, which are time-consuming and require a large computer memory especially for large data sets. Mozumder used SEER colorectal cancer data with more than 45,000 observations and showed that the SDH_FPM_2 runs fundamentally quicker than estimating the censoring weights and splitting the data [41]. Furthermore, SDH_FPM_2 is fitted on all three competing events simultaneously. However, if interest is in only one event there is no need to perform a model for all competing events and SDH_FPM_1 may be preferred [30]. One shortcoming of SDH_FPM_2 is that the model has some convergence problems with small sample sizes. Another problem with this model is that though the model fits for all competing events at the same time, there is no constraint on the sum of all cause-specific CIFs to be less than one [30]. This is also the case for SDH_FPM_1 because the models are fitted for each event separately [29, 30].

Using SDH_FPM_s, we investigated which covariates are associated with the risk of the first disease among HIV infected men. However, the effect of a covariate on the risk of an event can be misleading if not properly interpreted. Austin and Fine [47] conducted a review in medical papers, published in 2015, and showed that many of these papers had an incorrect interpretation of the results of the Fine & Gray model. In our study, the risk of AIDS prior to non-AIDS among patients with ≥ 40 age at diagnosis was less than < 40, whereas it has no significant effect on the occurrence of AIDS. This implies that the hazard of developing AIDS for a patient infected with HIV at the age of 60 is not statistically different from a patient infected at the age of 30. In a competing risks framework, in which the occurrence of the first event is raised, the unrelated death or non-AIDS diseases among ≥ 40 are more likely compared to < 40 and many elderly patients experience unrelated death or non-AIDS diseases prior to AIDS. So, fewer patients in ≥ 40 age group remain in the risk set of experiencing AIDS as the first event and the probability of AIDS occurrence as the first disease among them is reduced. As a result, we have implemented cause-specific hazards flexible parametric models in addition to subdistribution hazard flexible parametric models. We agree with the recommendation that both cause-specific hazards and subdistribution hazards-based models should be used for a comprehensive inference in competing risks analyses [10, 11]. Using CSH_FPM_ as well as SDH_FPM_, we could investigate the consistency of the effects of covariates on the hazards and risks of competing events and direct and indirect effects of them on the risks. The CSH_FPM_ can be performed for each event separately or as a unified model on the stacked data. The advantage of the unified model over separate CSH_FPM_s is that there is no need to implement FPMs on each event separately. However, using a unified CSH_FPM_ has some drawbacks. The unified model, by default, considers the same knot positions for all events. Also, the model is more complicated than separate CSH_FPM_ and convergence problems may occur.

Another important aspect of this study was investigating the occurrence of different types of events among HIV positive patients at the presence of each other. Recently, Pettit et.al [48]. showed that the risk of non-AIDS mortality among patients with an AIDS-defining event was twice higher than patients without an AIDS-defining event. However, most of the HIV studies were focused on AIDS and non-AIDS separately. Baker et.al [6]. examined the association of baseline CD4 cell counts on the AIDS and non-AIDS separately. They showed that the higher CD4 levels at baseline were associated with a lower risk of non-AIDS diseases (HR = .84, 95% CI (.74–.96)). However, considering an AIDS-prior event as a confounder, there was no significant association between baseline CD4 and non-AIDS diseases. This is consistent with our findings, which indicated that the risk of the occurrence of non-AIDS prior to AIDS is not associated with baseline CD4 levels at the presence of AIDS.

One of the limitations of this study was the challenge of Events-Per-Variable (EPV) for deaths prior to AIDS or non-AIDS disease, which may lead to sparse data biases and overfitting problem [49]. However, the main focus of this study was on AIDS and non-AIDS events. So, unrelated death was considered as a competing event that precludes the occurrence of AIDS/non-AIDS diseases. Another limitation was the lack of goodness of fit tests that be able to compare SDH_FPM_1 with SDH_FPM_2. SDH_FPM_s are newly developed approaches and probably the next step would be developing goodness of fit criteria for comparing them.

## Conclusion

This paper presents an application of flexible parametric models on multiple event types when each event alters the probability of occurrence of the other events. The choice of an appropriate model depends on the goals of the research and computational challenges. A review of these models and their required packages are summarized in Table 3.

**Table 3:** Approaches of Competing Risks Flexible Parametric Models

Model	Measures of associations	What is the model useful for?	How to model?	Advantages	Disadvantages	Which Stata commands?
Cause-specific hazard flexible parametric model	hazard	Etiological questions: which covariates have a causal effect on the occurrence of the event	CSH_FPM_	Easy to perform (on the original data) and interpret, using the standard FPM	Fitting separate models for each event	stpm2
			Unified CSH_FPM_	Fitting one model instead of separate models, using the standard FPM, Ability to handle shared covariate effects	Considering the same knot positions for all events, complex implementation (on the stacked data), Potential convergence problems	Stratified stpm2
Cause-specific subdistribution hazard flexible parametric model	Subdistribution hazard and cumulative incidence function (risk)	Prognosis questions: What fraction of patients are at risk to experience the event at a particular time	SDH_FPM_1	Fitting a separate model for the event of interest, using the standard FPM	Intensive computation (not ideal for large data sets), no constraint on the sum of CIFs	stcrprep and stpm2
			SDH_FPM_2	Fitting a unified model for all events (when the focus is on all events), Easy to perform (on the original data) and interpret, Less computation (ideal for large data sets)	convergence problems for small sample sizes, no constraint on the sum of CIFs	stpm2cr

### Supplementary information

**Additional file 1: Table S1.** Results of Transforming and Imputing Variables.

**Additional file 2: Table S2.** Results from the Fine & Gray Analysis of 10 Multiply Imputed Datasets.

## Data Availability

The datasets generated and/or analyzed during the current study are available in the Multicenter AIDS Cohort Study (MACS) repository, [http://aidscohortstudy.org/researchers/].

## Appendix

### sensitivity analysis

The data contained covariate missing values (7.6% of individuals had at least one missing value in their covariates). To address the impact of missing data, we used MI to examine whether the associations estimated in the standard analyses (Fine & Gray models) could have been biased due to exclusion of patients with missing data (CCA). Age at diagnosis, CD4, CD8, WBC, RBC, and platelets were registered for imputation. Survival outcomes consist of survival time and type of the event (as a factor with 4 categories for AIDS, non-AIDS, death and censoring), and MACS recruitment years were considered as predictors of the imputation models. In addition, we used hemoglobin as axillary information. For imputation, the multivariate normal regression method was used and *n* = 10 imputed data set were created. Univariate analyses on multiply imputed data sets were fairly close to CCA. Additional file 2: Table S2. includes the pooled results of final Fine & Gray models on the imputed data. The results are almost identical to CCA.

## Abbreviations

AIC: Akaike Information Criterion
AIDS: Acquired Immunodeficiency Syndrome
CCA: Complete Case Analysis
CIF: Cumulative Incidence Function
CR_FPM_: Competing Risks Flexible Parametric Model
CSH_FPM_: Cause-Specific Hazard Flexible Parametric Model
EPV: Events-Per-Variable
FPM: Flexible Parametric Model
HBM: Homosexual and Bisexual Men
HIV: Human Immunodeficiency Virus
LR: Likelihood Ratio
MACS: Multicenter AIDS Cohort Study
MCAR: Missing Completely at Random
MI: Multiple Imputations
SDH_FPM_: Subdistribution Hazard Flexible Parametric Model
