# Supplementary material for: Methods of competing risks flexible parametric modeling for estimation of the risk of the first disease among HIV infected men
Source: BMC Med Res Methodol. 2020 Jan 29;20:17. doi: 10.1186/s12874-020-0900-z (PMC6990537; doi:10.1186/s12874-020-0900-z)
Supplement: Supplementary file 1 — Additional file 1: Table S1. Results of Transforming and Imputing Variables. [file 12874_2020_900_MOESM1_ESM.docx]

**Additional file 2: *Table S2*. Results from the Fine & Gray Analysis of 10 Multiply Imputed Datasets.**

| Event | Variable |  | _SD_HR (95% CI) |
| --- | --- | --- | --- |
| AIDS | MACS recruitment |  |  |
|  | 1984-85 & 1987-90 |  | Reference |
|  | 2001-3 & 2010 |  | .030(.004–.21) |
|  | Age at diagnosis |  |  |
|  | <40 |  | Reference |
|  | $\geq$40 |  | .73(.54–.98) |
|  | Baseline CD4 |  |  |
|  | <350 |  | 1.67(1.06–2.62) |
|  | 350-500 |  | 1.41(1.03–1.93) |
|  | $\geq$ 500 |  | Reference |
| Non-AIDS | MACS recruitment |  |  |
|  | 1984-85 & 1987-90 |  | Reference |
|  | 2001-3 & 2010 |  | 3.17(1.93–5.21) |
|  | Age at diagnosis |  |  |
|  | <40 |  | Reference |
|  | $\geq$40 |  | 2.67 (1.83–3.88) |
|  | Baseline CD4 |  |  |
|  | <350 |  | .87(.45–1.70) |
|  | 350-500 |  | .54(.28–1.06) |
|  | $\geq$ 500 |  | Reference |
| Death |  |  |  |
|  | Age at diagnosis |  |  |
|  | <40 |  | Reference |
|  | $\geq$40 |  | 2.05(.85–4.93) |
|  | Baseline CD4 |  |  |
|  | <350 |  | .82(.10-6.65) |
|  | 350-500 |  | 1.54(.50-4.74) |
|  | $\geq$ 500 |  | Reference |
